# Nanotherapeutics for Meningitis: Enhancing Drug Delivery Across the Blood-Brain Barrier

**DOI:** 10.3390/biomimetics10010025

**Published:** 2025-01-03

**Authors:** Hitaishi Sharma, Kannan Badri Narayanan, Shampa Ghosh, Krishna Kumar Singh, Prarthana Rehan, Aparajita Dasgupta Amist, Rakesh Bhaskar, Jitendra Kumar Sinha

**Affiliations:** 1 GloNeuro, Sector 107, Vishwakarma Road, Noida 201301, Uttar Pradesh, India; 2 School of Chemical Engineering, Yeungnam University, Gyeonsang 38541, Republic of Korea; okbadri@gmail.com; 3 Research Institute of Cell Culture, Yeungnam University, Gyeonsang 38541, Republic of Korea; 4 Symbiosis Centre for Information Technology (SCIT), Symbiosis International (Deemed University), Hinjawadi, Pune 411057, Maharashtra, India; 5 Amity University Uttar Pradesh (AUUP), Sector 125, Gautam Buddha Nagar, Noida 201303, Uttar Pradesh, India

**Keywords:** biocompatibility, nanomedicine, meningitis, blood–brain barrier, liposomes

## Abstract

Meningitis is the acute or chronic inflammation of the protective membranes, surrounding the brain and spinal cord, and this inflammatory process spreads throughout the subarachnoid space. The traditional drug delivery methods pose a disadvantage in limiting the capacity of crossing the blood–brain barrier (BBB) to reach the central nervous system (CNS). Hence, it is imperative to develop novel approaches that can overcome these constraints and offer efficient therapy for meningitis. Nanoparticle (NP)-based therapeutic approaches have the potential to address the limitations such as penetrating the BBB and achieving targeted drug release in specific cells and tissues. This review highlights recent advancements in nanotechnology-based approaches, such as functionalized polymeric nanoparticles, solid lipid nanoparticles (SLNs), nanostructured lipid carriers, nanoemulsions, liposomes, transferosomes, and metallic NPs for the treatment of meningitis. Recently, bionics has emerged as a next-generation technology in the development of novel ideas from biological principles, structures, and interactions for neurological and neuroinfectious diseases. Despite their potential, more studies are needed to ensure the safety and efficacy of NP-based drug delivery systems focusing on critical aspects such as toxicity, immunogenicity, and pharmacokinetics. Therefore, this review addresses current treatment strategies and innovative nanoparticle approaches, and it discusses future directions for efficient and targeted meningitis therapies.

## 1. Introduction

Meningitis is a neurological disorder caused due to inflammation of the three-layered structure known as meninges, which protects the central nervous system (CNS). This inflammation disrupts various physiological processes, resulting in life-threatening conditions. The causes of meningitis can include a range of infectious agents such as bacteria, fungi, or viruses. These agents are generally classified based on factors including the time-dependent variations in cerebrospinal fluid (CSF) profiles, inflammatory reactions, and specific pathogens involved [1]. Meningitis can be either infectious or non-infectious in nature. Non-infectious meningitis may occur due to conditions such as neoplasms, autoimmune disorders, or drugs that provoke inflammatory responses [2]. Acute aseptic meningitis is the most common form of meningitis caused by enteroviruses [3]. Bacteria such as Streptococcus pneumonia and Neisseria meningitidis are generally causative agents resulting in bacterial meningitis [4]. Previous studies have shown that older individuals are more likely to be affected by meningitis due to a decline in immune function with age [5].

One of the major challenges associated with meningitis is its early diagnosis to detect early symptoms for better preventive measures. A traditional diagnostic method for meningitis involves CSF analysis, which assesses white blood cell (WBC) counts along with glucose and protein levels in the fluid [5]. The blood–brain barrier (BBB) serves as a crucial barrier against pathogens and foreign particles attempting to cross into the brain and plays a significant role in therapeutic interventions for meningitis and other neurological disorders such as Parkinson’s disease, epilepsy, and Alzheimer’s disease [6,7]. Along with the current treatments, nanotherapeutics present a promising avenue to overcome the obstacles associated with treating meningitis [8]. Various nanoparticles (NPs) such as lipids, liposomes, dendrimers, and magnetic NPs are used in clinical applications and are ideal substances that can perform targeted drug delivery by crossing the BBB [9]. The nanoparticle (NP)-based targeted drug delivery to the receptors is carried out using three major mechanisms, which are active targeting, receptor-mediated targeting, and transporter-mediated targeting. Recent studies have suggested nanotherapeutics as an emerging approach for treating neurological disorders, offering significant potential to overcome current limitations and paving the way for future advancements [10]. The primary complication in treating meningitis is the need to cross the BBB to target the CNS, which is inefficient with traditional drug delivery systems. To address these challenges, hydrogels and NP-based drug delivery methods are emerging as interventions with the ability to improve patient outcomes [11]. This article addresses the recent advancements and therapeutic interventions for treating meningitis using nanoparticle (NP)-based drug delivery systems.

## 2. Molecular Pathogenesis of Meningitis

**Figure 1:**
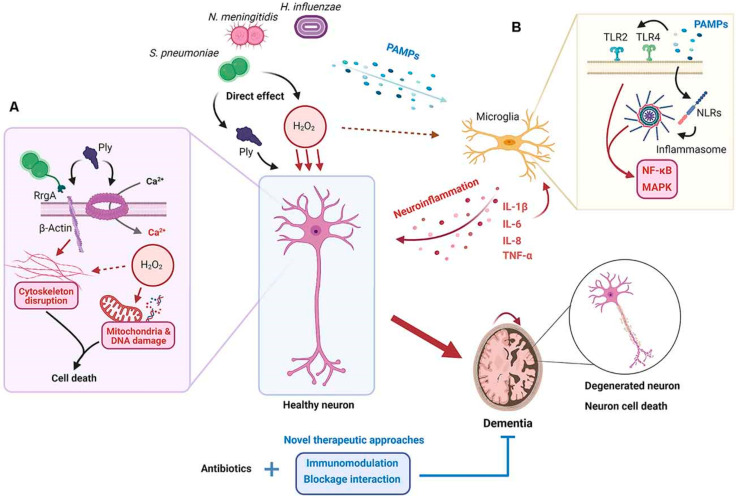
Mechanisms underlying neuronal damage in bacterial meningitis. When bacteria such as *Streptococcus pneumoniae*, *Neisseria meningitidis*, and *Haemophilus influenzae* infect the brain, their virulence factors and the resulting neuroinflammatory response contribute to neuronal damage and cell death. (**A**) Bacterial virulence factors, including pneumolysin (Ply) and pilus-associated RrgA, interact with neuronal components, leading to cytoskeletal disruption via β-actin interference and calcium influx. Ply also generates hydrogen peroxide (H_2_O_2_), which damages mitochondria and DNA, ultimately resulting in neuronal cell death. (**B**) Pathogen-associated molecular patterns (PAMPs) from the bacteria activate microglia via Toll-like receptors (TLR2 and TLR4) and nucleotide-binding leucine-rich repeat-containing receptors (NLRs). This activates the inflammasome and downstream signaling pathways such as NF-κB and MAPK, resulting in the production of proinflammatory cytokines (e.g., IL-1β, IL-6, IL-8, and TNF-α). The resulting neuroinflammation exacerbates neuronal damage, contributing to cell death and degeneration, which may lead to dementia. Therapeutic approaches combining antibiotics with immunomodulation and blockade of these pathological interactions are proposed to mitigate neuronal damage.

Meningitis is described as an inflammatory condition where the meninges protecting the brain are affected by bacterial or viral invasion leading to damage in brain parenchymal regions (Figure 1). Enteroviruses causing viral meningitis are classified with symptoms such as neck stiffness, dizziness, photophobia, and in severe cases, seizures [2,4]. The mode of entry for such invasive pathogens can be due to both direct and indirect contact (e.g., via respiratory droplets). Upon reaching the bloodstream, an immune response initiated by immunoglobulin (IgA) is induced to fight the pathogen [12]. Bacteria entering the bloodstream through the mucosal layer are attacked by the macrophages, which perform phagocytosis of the pathogens. If the pathogen survives this immune response, it reaches the CSF from the receptor sites. Once reaching the CSF, bacteria multiply rapidly in the subarachnoid cavity, which further initiates a response from receptors such as Toll-like receptors (TLRs) and T-cells [13]. Other contributing factors, such as tumor necrosis factor-alpha (TNF-α) and interleukin-1β (IL-1β), which are cytokines, further contribute to generating an inflammatory response [2,3].

## 3. Existing Therapeutic Interventions

Due to the life-threatening nature of meningitis, early diagnosis and treatment are crucial. Major therapeutic strategies focus on alleviating symptoms, such as reducing intracranial pressure [14]. The initial treatment for any form of meningitis typically involves empiric therapy, such as antimicrobial treatment. The secondary therapeutic approach involves the use of antibiotics and their dose and types depending on the severity of the disease. In addition, corticosteroids are generally used to decrease inflammatory activity. To inhibit an enterovirus and its activity, the pleconaril drug has shown promising results. Corticosteroid dexamethasone, when administered alongside antimicrobial treatment, has shown positive outcomes in patients [14]. Since oral administration is not approved for various drugs, vaccines provide another effective method for antiviral treatments, such as enterovirus D68 (EV–D68) vaccines and vaccine-like particle (VLP) vaccines [3]. Vaccines are also available to prevent the occurrence of meningitis. Menomune, Menhibrix, and Menveo are some vaccines used for children aged between 11 and 12 years. Antibiotic intervention is advised to be given within 24 h of diagnosis. Chemoprophylaxis antibiotics, including rifampin, ciprofloxacin, and ceftriaxone, are recommended for patients [14]. Recently, a nanoparticle (NP)-based vaccine modified with the chitosan PGLA NP molecule was formulated against *Escherichia coli*, which causes meningitis in mice. This vaccine mimics the structure of outer membrane protein A (OmpA), which is used by *E. coli* to disrupt the endothelial layers of the BBB. Experiments showed that chitosan-coated PGLA NPs successfully stabilized vaccines for meningitis [15,16]. Other nanoparticles, such as gelatin, polyglycolic acid, and polyalkyl cyanoacrylate, have also been investigated [2].

Nanogels are gels composed of nanoparticles that exhibit properties similar to NPs. Experiments have been conducted to treat bacterial meningitis using an intranasal administration of nanogels. Nanogels, also used as vaccines targeting pneumococcal surface proteins and attaching cationic entities, represent a potential area of research, yielding positive results that warrant further exploration [17]. Overall, various traditional therapies, combined with modern treatments, are being studied for meningitis. The growing field of vaccines and nanogel formulations show promise in preventing and treating major neurological disorders.

## 4. Problem and Role of BBB for Treating Meningitis

The primary challenge in treating neurological disorders like meningitis is the need to cross the BBB, which is structured to prevent the invasion of foreign particles. The BBB consists of endothelial cells and defense cells, with selective permeability. While the permeability of the BBB prevents pathogen invasion, the bacteria responsible for meningitis can cross this barrier, making treatment difficult. Therefore, drug-based therapies combined with nanoemulsions and NPs are needed to cross the BBB and deliver drugs directly to the brain. Currently, the most successful therapeutic approach is intranasal administration [18].

Bacterial meningitis occurs when the bacteria infiltrate the BBB, often accompanied by significant bacteremia. Understanding host factors that facilitate bacterial transmission to the BBB is crucial for developing strategies to treat bacterial meningitis [19]. NP-based therapies for meningitis are attributed to their increased surface-to-volume ratio and structural adaptations, which provide improved penetration of the BBB. However, the administration of NPs for drug delivery can have severe side effects, such as brain damage or functional difficulties.

One method to cross the BBB involves the disruption of the endothelial layer, which is induced by the osmotic agent mannitol. Although it is combined with NPs to improve the drug’s delivery, mannitol poses significant risks of adverse effects on brain tissues, causing abnormal functioning or damage. Crossing the BBB remains a major challenge in treating brain disorders, but NPs have shown promise as a potential intervention for neurological diseases [20]. Therefore, while the BBB is a significant obstacle in treating meningitis, the use of nanoparticles, micelles, or nanoemulsions is emerging as a potential solution for delivering drugs directly to the brain. However, further research is needed to minimize the risks and side effects associated with these treatments. In order to enhance treatment strategies, investigating host activity and the underlying pathways for bacterial transmission to the BBB represents a promising area of study with significant potential outcomes.

## 5. Nanoparticle-Based Approach for Neurological Disorders

When designing nanocarriers for medicinal applications, it is important to consider several key features such as drug targeting specificity, reduced toxic effects associated with nanoparticle uptake, biocompatibility, and innovative approaches for developing new therapies. NP-based drug delivery systems offer several advantages over traditional therapeutic methods. They can be engineered to enhance properties such as solubility, drug stability, and bioavailability. Moreover, the controlled and sustained release of drugs can be tailored to minimize side effects. The specific targeting of cells is vital to minimize damage to neighboring tissues, especially when treating conditions like cancer. Overcoming this challenge is essential for improving the efficacy of NP-based therapies [21]. Another approach includes NP-induced immune response. Modifying the surface of NPs can enhance their ability to penetrate the CNS [22]. Despite the limitations and potential side effects associated with NP-based therapies, they represent promising candidates for treating neurological disorders.

### 5.1. Classification of Nanoparticles

**Figure 2:**
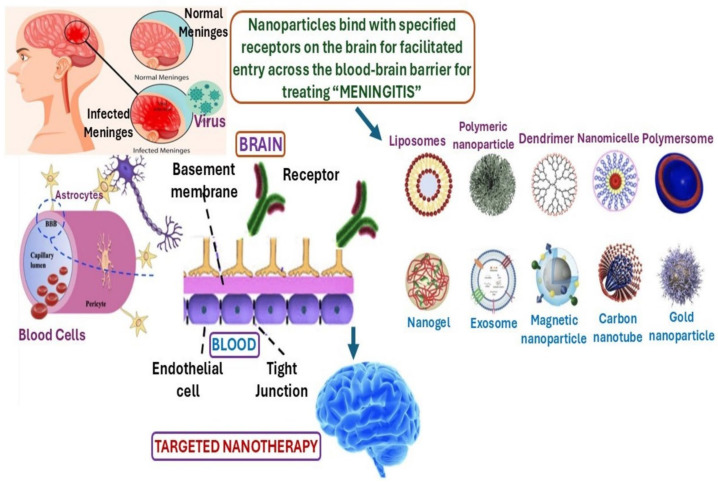
Neurological disorder treatment with various nanoparticle-based therapeutics. Reproduced from an open-access image published by MDPI publishers [27].

Nanoparticles can be categorized based on their composition, shape, size, and surface charge. Based on composition, they are classified into two types, i.e., organic and inorganic NPs. Organic nanoparticles include polymeric nanoparticles, lipid-based nanoparticles, dendrimers, and micelles, while inorganic nanoparticles include metal nanoparticles, quantum dots, and carbon nanotubes (Figure 2) [23]. Based on shape, NPs can be classified into rod-shaped, spherical, or disk-like structures. They can vary in size, being ultra-small (<10 nm), small (10–100 nm), or large (>100 nm). Each type of NP has distinct features and limitations. Liposomes are biocompatible due to their phospholipid structure and vesicles filled with cholesterol particles, which can carry both hydrophilic and hydrophobic drugs [24]. Polymers are used for their higher stability and drug-binding capacity [25]. Solid lipid nanoparticles (SLNs), a type of organic nanoparticle, are composed of a lipid core surrounded by a surfactant layer, and they are known for their stability and biodegradability [26].

#### 5.1.1. Polymer-Based Nanoparticles (PBNPs)

Polymer-based NPs (PBNPs), ranging in size from 60 to 200 nm, are potential candidates for drug delivery. The hydrophilic corona of these nanoparticles contributes to their steric stability and prevents aggregation in physiological fluids [26]. Nanoparticle-incorporated polymers such as polyglycolide (PGA), polylactides (PLA), and chitosan are commonly used for drug delivery [25,28]. To enhance the drug delivery efficacy of PBNPs, the surface of NPs can be modified with hydrophilic polymers, either through covalent bonding or physical adsorption to increase their residence time in the bloodstream. Additionally, conjugating tissue-specific ligands to these functionalized polymers or directly to the nanoparticle surface can enable targeted delivery to specific tissues or organs. Studies have shown that the permeability of drugs can be enhanced by loading higher quantities of polymer nanocarriers, increasing the overall efficacy of the drug [29]. PBNPs have tremendous potential as drug delivery agents and can play an important role in developing new therapeutic strategies for neurological disorders. Dendrimers, which are characterized by their highly controlled shape and size and well–defined structures, possess branch–like architectures known as hyperbranched generations. These branches provide surfaces for attaching functional groups, enhancing the efficiency of ligand functionality. The high solubility, biodegradability, and biocompatibility of dendrimers make them ideal candidates for safe biomedical treatments [30].

The surface of dendrimers can be modified for bonding with targeting ligands such as antibodies and peptides, increasing specificity for targeted drug delivery [31]. Moreover, the multivalency of dendrimers enables the simultaneous delivery of multiple drugs or diagnostic agents, making them advantageous for combination therapy or imaging applications. Moreover, polymeric micelles have shown promising results as drug delivery carriers due to their ability to encapsulate hydrophobic drugs, increasing their solubility and bioavailability [32]. The hydrophilic shell of polymeric micelles shields the hydrophobic core, providing stability and protection against opsonization by the immune system. These polymeric micelles can also be functionalized with targeting ligands to increase their specificity for cancer cells or other targeted tissues. Polymeric micelles have been investigated for the delivery of a wide range of therapeutic agents, including small molecules, proteins, and nucleic acids [33], and can deliver multiple drugs in a single formulation [34]. Therefore, both dendrimers and polymeric micelles show great potential for drug delivery due to their unique physicochemical properties, ease of functionalization, and ability to encapsulate a variety of drug molecules. Their biocompatibility and biodegradability properties make them attractive options for biomedical applications, particularly in targeted drug delivery.

#### 5.1.2. Lipid-Based Nanoparticles (LBNPs)

Lipid-based nanoparticles (LBNPs) have attracted considerable attention as nanocarriers for delivering drugs because of their distinct characteristics. Liposomes, a type of nanocarrier composed of one or more vesicular lipid bilayers called lamellae and made of biodegradable and biocompatible lipids with amphiphilic properties, can encapsulate both hydrophilic and hydrophobic drugs and have been intensively used for delivering drugs to the brain [29]. Cationic liposomes, which contain positively charged lipids, are used for transporting genetic material and as delivery vehicles. DOTAP, combined with DOPE, is one of the most commonly used cationic liposomes for drug delivery. Research has demonstrated that cationic liposomes carrying photosensitive drugs can induce the laser-triggered cytotoxic effects on glioblastoma cells and can enhance the delivery of paclitaxel, a mitotic inhibitor used in cancer chemotherapy.

##### Solid Lipid Nanoparticles (SLNs)

Solid lipid nanoparticles (SLNs) are a type of LBNPs typically spherical and ranging from 50 to 100 nm in size, showing higher biological stability and lower toxicity than liposomes [28,35,36]. SLNs are lipid molecules that remain solid at room temperature. The composition of SLNs typically includes triglycerides, fatty acids, or waxes, and their solid lipid core (hydrophobic) serves as a site for drug dissolution or dispersion. Due to their small size, SLNs can traverse the tight endothelial cells of the BBB and evade the reticuloendothelial system (RES). This characteristic enables them to function as effective drug delivery systems for the brain. Various types of lipids, including glyceryl behenate, trimyristin, and stearic acid, have been utilized for SLNs with positive outcomes for medical applications. Advantages of SLNs as nanocarriers include sustained drug release over weeks, the ease of surface modification for drug targeting, and reduced uptake by the RES [34]. SLNs have been successfully used for oral drug delivery, particularly for neurological disorders. SLNs have facilitated an increased oral administration of drug carriers for patients with neurological disorders, allowing for passage through the stomach and intestinal mucosal layers. They have shown effectiveness in patients with conditions such as Alzheimer’s disease, stroke, or depression through the oral administration of microemulsions coated with curcumin. It was reported that as the brain has structures composed of a phospholipid layer, the lipid components of SLNs play a significant role in inducing excitability in the brain [37,38].

##### Nanoliposomes (NLPs)

Nanoliposomes (NLPs) are a type of nanosized carrier system that can effectively deliver drugs to the brain due to their unique structure, which consists of a lipid bilayer that can encapsulate hydrophilic or hydrophobic drugs. These biodegradable molecules have lower toxicity and can be administered intravenously [2]. The first FDA-approved nanoliposome drug, Doxil, has been used to treat various cancers, and a drug called Nanotherm is currently in trials for prostate cancer treatment [39]. Notably, cationic liposomes have demonstrated potential for brain-targeted drug delivery, with the addition of sulfur nanoparticles shown to increase their ability to penetrate the BBB [40]. Surface modifications of liposomes address the complications associated with BBB permeability. A study demonstrated the development of a brain-targeted drug formulation consisting of the antidepressant sertraline coated with glycosylated and pegylated liposomes, resulting in improved brain delivery [21]. Beyond their applications in brain disorders, liposomes have also been employed to target fungal and bacterial infections in the spleen and liver [41]. Overall, liposomes exhibit significant potential for targeted drug delivery in various neurological disorders, including meningitis. However, optimizing drug delivery and improving liposome properties are required to mitigate challenges related to toxicity and immunogenicity [42].

##### Nanoemulsions (NEs)

Nanoemulsions (NEs) are emulsions with sizes ranging from 20 to 200 nm, exhibiting enhanced stability and transparency. They are composed of two immiscible liquids, typically oil and water, stabilized using surfactants and/or co-surfactants. NEs are thermodynamically stable and possess a high surface area, making them suitable for various applications in drug delivery, cosmetics, and the food industry [43]. Due to their small droplet size, NEs exhibit improved bioavailability, greater stability, and drug release properties compared to traditional emulsions. They can encapsulate both hydrophobic and hydrophilic molecules and target specific tissues, such as the brain, by crossing the BBB [44]. NEs have shown potential in treating diseases such as cancer, Alzheimer’s disease, and other neurological disorders, as well as fungal infections with various administration routes, including oral, intranasal, intravenous, and topical routes [45]. Moreover, they can be formulated into different forms, such as sprays, creams, gels, and liquids for targeted drug delivery in neurological disorders, including meningitis [2]. NEs can exist as biphasic (water-in-oil or oil-in-water) or multiphasic systems, depending on the components used. Risperidone-loaded and saquinavir-loaded biphasic NEs have been proposed as effective drug carriers for brain-targeted drug delivery due to their ability to permeate the BBB [46]. Advancements in the improvement of stability, biocompatibility, and targeted drug delivery in NEs for enhanced neurological therapy will increase their clinical potential [20].

#### 5.1.3. Metallic Nanoparticles (MNPs)

Metallic NPs (MNPs) are small particles ranging from 10 to 100 nm in size. These NPs can be made up of metals like copper, silver, and/or gold, which have been used in therapeutics for centuries. Among these, silver NPs (AgNPs) are highly versatile and used in the management of neurological diseases due to their diverse physical and chemical properties. In addition, gold NPs (AuNPs) were historically used in Ayurvedic medicine in ancient India to treat various nervous system disorders [47]. AuNPs with a colloidal gold size of 56 nm, known as Swarna bhasmal (gold ash), were mixed with honey or cow ghee and administered orally to treat various diseases, including neurological disorders [48].

Today, MNPs are extensively used in cancer therapy, antibacterial treatments, and theranostic applications. The advantages of MNPs include their large surface area-to-volume ratio, which enhances cellular uptake and makes them suitable for targeted drug delivery applications [49]. Moreover, MNPs are cost-effective, making them a suitable alternative for pharmaceutical applications [50]. Despite their promise, potential toxicity remains a major concern, especially when it comes to brain targeting [48]. MNPs have shown potential for targeted drug delivery to the brain for neurological disorders such as glioma and meningitis [51]. To address toxicity issues, researchers are exploring various methods to enhance the biocompatibility and safety of MNPs in neurological treatments. One primary strategy involves surface modification, where MNPs are coated with biocompatible polymers, such as polyethylene glycol (PEG), which decreases interactions with biological membranes and immune cells, thereby lowering the risk of oxidative stress and inflammation [52,53]. PEGylation has been shown to improve the biocompatibility of AuNPs and AgNPs while enhancing their circulation time in the bloodstream [52,53]. Another approach involves the optimization of MNP particle size and shape to minimize tissue accumulation while facilitating excretion. Previous studies have indicated that smaller MNPs (<20 nm) are more readily excreted through renal clearance, reducing the risk of accumulation in vital organs [54]. Spherical and rod-shaped MNPs, due to their controlled surface interactions, have been shown to exhibit lower toxicity than irregularly shaped MNPs [54]. Functionalization with biocompatible molecules and antioxidants are an effective method to mitigate the toxicity of MNPs, as conjugating them with molecules such as glutathione or curcumin can neutralize reactive oxygen species (ROS) generated by the MNPs, thereby reducing oxidative damage [55]. Finally, the dosage optimization and route of administration of MNPs play a critical role in minimizing neurotoxicity. Using targeted delivery systems, such as ligand-based targeting to specific receptors, ensures that MNPs accumulate primarily at intended sites, minimizing off-target effects [56]. These strategies, alongside ongoing research into the long-term effects of MNPs, are essential for the safe and effective implementation of these MNPs in clinical settings for treating neurological disorders [57]. Recent studies have also explored other promising approaches for brain targeting using MNPs. For instance, researchers have investigated the use of ultrasmall AuNPs (2 nm) that can traverse the in vitro BBB in a six-cell human brain three-dimensional (3D) spheroid model [58]. Furthermore, the intranasal administration of iron oxide NPs has been shown to facilitate their migration to the brain along the olfactory nerve, effectively bypassing the BBB [59]. These advancements, combined with further research into the long-term effects of MNPs, will be pivotal in ensuring their safe and effective clinical applications for targeted drug delivery in neurological disorders.

## 6. Properties of Nanoparticles as Drug Delivery Vehicles Across Blood–Brain Barrier

**Figure 3:**
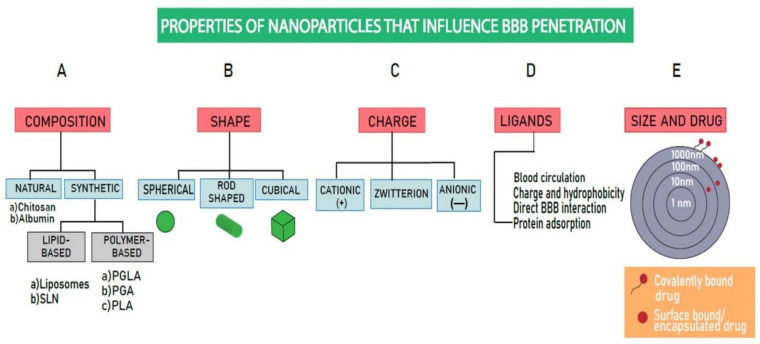
Nanoparticle properties influencing BBB penetration. Various factors of nanoparticles affect their penetration through the BBB. (**A**) Nanoparticles can be natural (e.g., chitosan, albumin) or synthetic, which can be further divided into polymer-based nanoparticles (PGLA, PGA, PLA) or lipid-based nanoparticles (nanoliposomes, SLNs). (**B**) Nanocarriers can vary in shape (spherical, rod-shaped, or cubical). Spherical NPs are widely used in various neurological treatments. (**C**) The surface charge (positive, negative, or neutral) influences their ability to penetrate the BBB. Positively charged NPs are more readily taken up by cells in comparison to neutral and negatively charged NPs. (**D**) Another factor determining the penetration of NPs through the BBB is the nature of ligands attached to them, which can be categorized based on their (1) ability to enhance the circulation of blood, (2) ligands that increase hydrophobicity, (3) ability to directly interact with BBB receptors, and (4) role in regulating protein adsorption. (**E**) Based on the size of NPs (1–1000 nm), encapsulated drug, or surface-adsorbed drug either ionically or covalently. Abbreviations: BBB—blood–brain barrier; PGA—polyglycolide; PGLA—poly lactide glycolic acid; SLN—solid lipid nanoparticle; nm- nanometer.

Nanoparticles are also a type of nanocarrier serving as delivery vehicles for various substances, such as therapeutic and diagnostic agents. The blood–brain barrier (BBB) is the physiological barrier between the CNS and the bloodstream that controls the movement of substances into the brain [60]. This barrier plays a vital role in protecting the CNS by regulating the entry of harmful foreign molecules, thereby maintaining CNS homeostasis, enabling proper functioning of the neuronal system and protecting the neural tissue from pathogens and other foreign particles [61,62]. Several factors influence the permeability and efficacy of the nanoparticle-mediated drug delivery systems across the BBB. Factors such as nanoparticle size, shape, charge, and nature of ligands attached to their surfaces can be optimized to enhance bioavailability and BBB permeability (Figure 3).

### 6.1. Size and Shape

Size is one of the crucial factors that influence the permeability of nanocarriers across the BBB. Nanoparticles of different sizes exhibit varying properties such as circulation half-time and extravasation through leaky vasculature. Nanocarriers with sizes ≤ 200 nm are preferred because they exhibit deeper brain penetration and longer circulating times compared to larger nanocarriers. However, smaller nanoparticles have limitations, such as lower encapsulation efficiency and rapid drug release. Nanoparticles smaller than 5 nm can easily be rapidly excreted out by the renal system. Therefore, the optimum size of brain-targeting nanocarriers is approximately 20 nm; this size facilitates efficient BBB penetration and reduces the risk of renal clearance [60,62]. Interestingly, the shape of nanoparticles also significantly affects systemic circulation, BBB passage, and cellular uptake. Spherical NPs are widely used due to the ease of large-scale production; however, their curvature limits the binding sites with membrane receptors. On the other hand, rod-shaped nanoparticles exhibit greater receptor interactions compared to spherical-shaped nanoparticles. Other geometric configurations, such as disk-like and ring-like NPs, have been shown to possess efficient targeting and penetrating properties compared to their spherical counterparts [63].

### 6.2. Surface Charge

The surface charge, or zeta potential, of a nanoparticle, also plays a vital role in its transport across the BBB and can significantly affect circulation lifetime. Moreover, the surface charge influences the aggregation and localization of drugs at a specific site of interest. Due to the negatively charged nature of the cell membrane, positively charged NPs can be easily internalized by cells compared to neutral or negatively charged NPs [63]. However, neutral and negatively charged NPs exhibit lower serum protein absorption, which leads to longer circulation half-times. At low concentrations, negatively charged (anionic) and neutral NPs do not compromise the integrity of the BBB. However, positively charged (cationic) NPs and high concentrations of negatively charged NPs have the potential to disrupt BBB integrity. Thus, when designing a nanocarrier for drug delivery to the brain, it is crucial to consider the effects of surface charge, protein absorption, and interaction with the BBB. An important consideration is that the initial surface charge does not always determine the fate of the NP; upon protein adsorption, a shift toward a net negative zeta potential can occur due to the formation of a protein corona [64,65].

### 6.3. Ligands

Ligands are molecules that bind to a receptor, which can trigger or regulate signaling pathways. The interaction between a ligand and its receptor is specific and selective. The limited targeting ability of NPs has led to challenges in the effective delivery of drugs across the BBB, resulting in poor therapeutic outcomes in neurological disorders. To overcome this issue, various targeting ligands are covalently conjugated or absorbed onto the NPs. The ligands that aid BBB penetration can be classified into four types, namely (a) ligands that increase blood circulation time, (b) ligands that enhance surface charge and hydrophobicity, (c) ligands that directly interact with the BBB, and (d) ligands that regulate the absorption of proteins that can interact with the BBB [63]. The primary mechanism for targeting involves the identification and selective binding of the ligand to the target substrate, such as the binding of targeting ligands to receptors overexpressed in diseased tissues. For instance, the transferrin receptor (TfR) is overexpressed in brain tumor cells as well as the BBB [66,67]. NPs with TfR targeting ligands can bind to Tf receptors on the BBB and facilitate the aggregation of the drug enclosed in the NP, thereby improving the therapeutic efficacy against the tumor-promoting factors [66].

### 6.4. Safety and Biocompatibility of Nanoparticles

Nanoparticle-based drug delivery systems have emerged as a promising approach for treating neurological disorders, including meningitis, by overcoming challenges associated with crossing the BBB; however, toxicity and biocompatibility remain critical considerations. NPs can induce toxicity through mechanisms such as oxidative stress, inflammation, and disruption of cellular homeostasis. For example, MNPs like AgNPs and AuNPs have demonstrated dose-dependent toxicity in preclinical animal models, with AgNPs causing oxidative damage and apoptosis at concentrations exceeding 20 mg/kg [49]. Despite their therapeutic potential, LBNPs can also exhibit moderate cytotoxicity under certain conditions [39]. These findings underscore the necessity for comprehensive safety evaluations during nanoparticle development. Immunogenicity is another critical challenge, particularly for nanoparticles engineered with surface modifications to enhance BBB permeability. Cationic NPs, for instance, can interact with negatively charged cellular membranes, potentially triggering immune responses, including hemolysis and inflammation [68]. Additionally, the formation of a protein corona—an adsorption layer of biomolecules on the NP surface—can modify their surface characteristics, leading to unintended immune activation and reduced therapeutic efficacy [69]. To address these concerns, strategies such as surface modifications have been employed; for instance, PEGylation has shown promise in minimizing immunogenicity by shielding NPs from protein adsorption and immune recognition [70,71]. However, optimizing nanoparticle size, charge, and surface chemistry to achieve a balance between efficacy with safety remains a persistent challenge.

## 7. Drug Delivery to Brain

Nanotherapeutics encompasses a wide range of techniques using nanoparticles for the treatment of neurological disorders. Although the BBB in the brain obstructs the passage of drugs or molecules into the CNS, various nanocarriers with specific modalities are under exploration for the treatment of brain disorders. Delivery to the cerebral region of the brain can be achieved through both invasive and non-invasive techniques, such as intracerebral and intranasal drug delivery systems. Additionally, the permeability of the BBB can be enhanced through specific interactions with growth factors or nutrient molecule-coated nanoparticles/nanocarriers. The nanocarrier properties that influence its brain delivery by crossing the BBB include size (100–300 nm), morphology, and targeted ligand–receptor interactions [72]. Different types of nanocarriers, including lipid nanocarriers, dendrimers, polymeric nanoparticles, and liposomes, are explored based on their unique characteristics for effective brain drug delivery across the BBB. Active targeting, also known as transcytosis, of the drug delivery system can be absorptive-mediated, transporter-mediated, and receptor-mediated transcytosis. These nanocarriers are specifically engineered to facilitate targeted delivery to the brain [72].

Lipid-based nanoparticles (LBNPs), such as solid lipid nanoparticles (SLNs), exhibit higher stability compared to polymeric NPs [73]. SLNs are also considered less toxic due to their biodegradable and biocompatible properties [26,73,74]. In contrast, polymeric NPs can exhibit varying levels of toxicity depending on their polymeric surface charge [73]. Nanometer-sized semiconductor quantum dots (QDs) are used as tools in brain nanotheranostics, but they are toxic due to the leaching of inorganic ions from the QDs [75]. On the other hand, platinum (Pt) NPs are generally considered non-toxic compared to AgNPs [76,77]. AuNPs and PBNPs are effective for therapeutic purposes, but their efficacy depends on the employed drug delivery mechanism [72,78,79]. Regarding the biodistribution and inflammation of therapeutic nanocarriers, studies have shown that combination with corticosteroid dexamethasone can reduce the acute inflammatory response to small-interfering-RNA-encapsulated SLNs (siRNA-SLNs) [80,81]. In addition, the severity of inflammatory toxicity of the nanoparticles is likely to vary depending on the drug-targeted organ. In terms of drug delivery, SLNs showed better efficacy in crossing the BBB due to their lipophilic nature, making them suitable for delivering drugs to the CNS [26]. SLNs have also been studied for delivering a variety of drugs, including anticancer drugs and antidepressants [42]. Overall, references suggest that LBNs, especially SLNs, have higher stability and lower toxicity compared to polymer NPs, while the toxicity of metal-based nanoparticles is size-dependent. The effectiveness of nanoparticles for drug delivery may be influenced by factors such as the nature of the drug delivery mechanism, target organs, and the use of combination treatments such as dexamethasone to alleviate the inflammatory mechanism.

### 7.1. Absorptive-Mediated Transcytosis (AMT)

Absorptive-mediated transcytosis (AMT) occurs through non-specific interaction with the cell surface, where nanoparticles/nanocarriers are internalized by cells through electrostatic interactions between particles with opposite charges and the endothelial layer of the BBB luminal surface. In this process, AMT follows an absorptive mechanism, and transcytosis refers to the transportation of nanoparticles across the cellular barrier via endocytosis. AMT is not cell-specific and is widely used for drug delivery applications. Clathrin-mediated endocytosis, a type of general endocytosis, is one of the common pathways involved in AMT, where nanocarriers can bypass the tight junctions between the endothelial cells of the BBB to deliver drugs into the brain. Additionally, molecules such as lectins, cardiolipin, heparin, and certain peptides are often used to enhance the AMT mechanisms for drug delivery by nanocarriers [72].

### 7.2. Receptor-Mediated Transcytosis (RMT)

**Figure 4:**
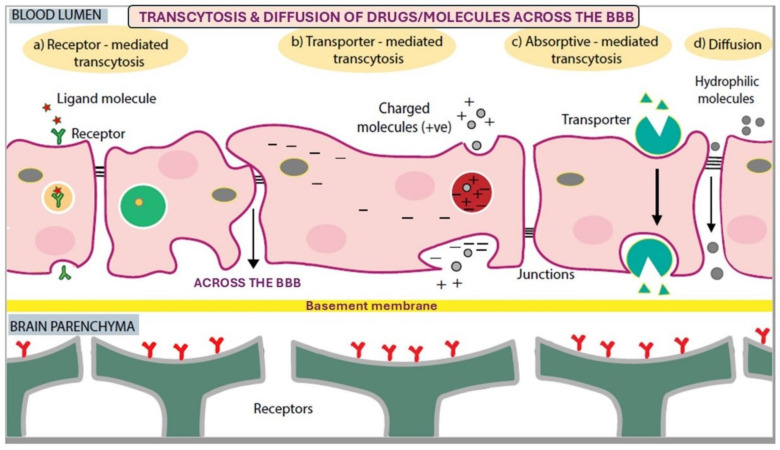
Drug delivery system across the BBB. Nanoparticles are transported through drug delivery systems that follow four types of mechanisms. (**a**) Receptor-mediated transcytosis, in which ligands (transferrin, low-density lipoprotein, insulin, and vitamins (folate, biotin)), coated NPs binds to their respective receptors, facilitating the nanoparticle transportation across the BBB; (**b**) transporter-mediated transcytosis is carried out by a transporter molecule, which carries the NPs across the BBB; (**c**) absorptive-mediated transcytosis, where the transport of NPs occurs between the electrostatic interactions between NPs and the endothelial cells of the BBB luminal surface; and (**d**) the diffusion of hydrophilic and lipophilic molecules occurs through the paracellular or transcellular, respectively, across the BBB. Abbreviations: BBB—blood–brain barrier; +ve—positive.

In receptor-mediated transcytosis (RMT), nanoparticles are transported to the brain via specific receptors on the BBB. The binding of a ligand with its receptor triggers endocytosis, followed by transcytosis [50]. Receptors such as lactoferrin, transferrin, and nicotinic acetylcholine are commonly used in the RMT pathway. Upon ligand–receptor interaction, the cell membrane invaginates to form a clathrin-coated pit, initiating clathrin-mediated endocytosis. Alternatively, other internalization mechanisms, such as caveolae-mediated endocytosis or non-clathrin endocytosis, may facilitate the internalization of the receptor–ligand complex. Subsequently, the vesicle releases its cargo into the brain through transcytosis [72] (Figure 4).

### 7.3. Transporter-Mediated Transcytosis (TMT)

Transporter-mediated transcytosis (TMT) is a specific type of transcytosis that facilitates the delivery of drugs across the BBB via membrane transporters. Generally, TMT plays a crucial role in transporting molecules such as nutrients, drugs, and other therapeutic agents across the BBB through transporter-mediated mechanisms. Different types of transporters involved in the transport of nanocarriers include glucose transporters (GLUTs), glutathione (GSH) transporters, amino acid transporters, and peptide transporters (PEPT1, PEPT2) [82]. Nanocarriers engineered with natural substrates such as amino acids, glucose, or peptides must interact with specific transporters on the endothelial cells of the BBB to cross the barrier and deliver drugs to the brain.

## 8. Intranasal Drug Delivery to the Brain

The intranasal route is the non-invasive and most effective way for drug delivery directly to the brain compared to oral or vascular routes. Drugs administered through the nasal cavity enter the circulatory system directly through the nasal mucosa. The nasal route offers significant benefits, including a non-invasive technique that allows for easy dose adjustments and rapid absorption due to its larger surface area. For instance, a formulation combining poly(lactic-co-glycolic acid) PGLA and SLN was administered intranasally for the treatment of Alzheimer’s disease [21]. There are three pathways recognized for nose-to-brain delivery, namely olfactory, trigeminal, and systemic pathways. The drugs can pass through the BBB via these routes and access the brain tissue or cerebrospinal fluid (CSF) [2]. The olfactory pathway is the primary route for nasal transmission, where drugs cross the epithelial layer through assisted movement, passive diffusion, endocytosis, or paracellular movement. In the trigeminal pathway, drugs reach the brain tissue via the trigeminal nerve, while in the systemic pathway, drugs pass through the BBB and access CSF.

The olfactory pathway is particularly significant, as it allows for the passage of drugs into the subarachnoid space through the olfactory nerve, distributing drugs across different brain regions. The major limitation of nasal route administration is the mucociliary defense mechanism, which protects the lungs from pathogens and prevents damage to the nasal mucosal layer [83]. Despite these challenges, intranasal drug delivery is potentially used to treat various neurological disorders such as Parkinson’s disease, multiple sclerosis, and migraine [84,85]. In addition to drug molecules, intranasal delivery can also be used for gene therapy delivery to the brain, showing potential in preclinical studies. The effectiveness of intranasal drug delivery can be improved using mucoadhesive polymers, permeation enhancers, and nanocarriers [86]. Intranasal drug delivery has shown promise in the treatment of acute ischemic stroke by delivering the drug directly to the brain to prevent or minimize brain damage [85,87]. Additionally, the intranasal route has also been used for vaccine delivery due to the presence of nasal mucosa’s immune cells, which can initiate effective immune responses against the antigen [88]. However, ensuring the safety of intranasal drug delivery is crucial, and precautions must be taken to minimize the risks of infection, irritation, and the systemic absorption of drugs through appropriate formulation and dosing strategies.

## 9. Bionics in Meningitis Treatment: A Transformative Approach

Bionics is a cutting-edge technology that significantly improves the lives of patients suffering from various ailments. It involves the comprehensive study of biological structures, principles, behaviors, and interactions among biological systems, aiming to generate novel design ideas and develop advanced technologies for humans. Rather than imitating biological structures, bionics focuses on the implementation of natural functions [89]. Recently, bionics has emerged as a transformative approach in the treatment of meningitis, facilitating not only its treatment but also early detection and diagnosis through various implantable devices and wearable sensors that monitor vital signs and cerebrospinal fluid (CSF) biomarkers or detect pathogens in real-time. Advanced applications of bionics include the development of a bioelectrode genosensor designed to detect the genomic DNA of Neisseria meningitidis, the major etiological agent of meningococcal meningitis within the human serum. Moreover, nanobionic systems have enabled the targeted drug delivery of antibiotics or anti-inflammatory drugs directly at the infection site, reducing systemic adverse effects. These systems, enhanced by microfluidic implants, can regulate the drug dosages and release timings to achieve optimal therapeutic outcomes. For instance, the “nanobionic liposome”, which mimics cell membranes at the subcellular scale, improves drug delivery efficiency through facilitated interfacial adhesion and tissue penetration [90]. Furthermore, Wu et al. (2021) developed a therapeutic agent of platelet and tumor cell membrane-camouflaged β-mangostin-loaded NPs for targeted delivery and anticancer activity against glioma, demonstrating efficacy in both in vitro and in vivo models by crossing the BBB [91]. This exemplifies the potential of NP-based systems and nanorobots to mimic or enhance natural biological mechanisms for precise and efficient drug delivery.

Moreover, the application of nanotechnology in the form of bionic scaffolds represents a significant advancement in therapeutic approaches for neurological disorders and neuroinfectious diseases. These scaffolds can be engineered to navigate the BBB and deliver therapeutic molecules directly to infected tissues within the CNS. Recent studies have demonstrated that magnetically guided nanorobots can traverse the BBB, enhancing drug delivery efficiency for neurological disorders, including meningitis. The integration of bio-inspired designs in nanotechnology has also led to the creation of nanorobots capable of navigating the complex biological environment of the CNS [92]. These nanorobots, programmed for magnetic guidance, can deliver drugs precisely to the target site [93,94]. Their ability to cross the BBB effectively opens promising avenues for treating meningitis and other CNS disorders. The application of bionics in treating meningitis through advanced drug delivery systems offers a multifaceted approach to overcoming the challenges posed by the BBB. By leveraging functionalized nanoparticles, inflammation-activated delivery systems, and nanorobots, researchers are developing innovative strategies to enhance therapeutic efficacy while minimizing adverse effects [93]. These advancements not only offer significant potential for enhancing treatment outcomes in meningitis but also establish a groundwork for broader applications in the management of various neurological disorders.

Addressing the long-term complications of meningitis and other neurological disorders involves the development of next-generation brain/neural computer interface technology. These bionic neural interfaces aim to restore lost functions by mimicking biological functions. Moreover, brain–machine interfaces (BMIs) support the rehabilitation of meningitis patients by facilitating communication between the brain and external devices or aiding cognitive recovery [95]. For meningitis patients with sensorineural hearing loss, bionic cochlear implants bypass damaged parts of the auditory system, thereby restoring hearing [96].

## 10. Limitations **and Challenges** of Nanotherapy

Nanotherapy has emerged as a breakthrough approach to treating various diseases, including neurological diseases [97]. However, widespread clinical applications have several limitations and challenges that need to be addressed for effective implementation [98,99]. Smaller NPs may cause both cellular and systemic-level toxicity. The potential toxicity of NPs is a significant concern, as their interaction with the immune system creates physiological barriers, which is a major issue when administered (Table 1). Many NP formulations, especially those utilizing lipid-based nanocarriers, are prone to decomposition or aggregation under physiological conditions, which reduce their efficacy and alter biological distribution [100,101]. One of the biggest challenges in using NPs is ensuring their stability. While surface modification and encapsulation strategies have been used to address these issues, maintaining long-term stability during storage and after administration remains a significant challenge. Many NPs, especially LBNPs, are prone to aggregation and decomposition in physiological environments, which can impair efficacy [36]. For example, SLNs have improved stability over other LBNPs, but long-term storage and transport are still a problem due to their sensitivity to temperature and pH [26,35,73]. Scalability is another important limitation. The transition from laboratory-scale synthesis to large-scale manufacturing is often challenging because the nanoparticle properties are sensitive to production conditions such as mixing, temperature, and solvent interactions [32,102]. This variability can lead to inconsistent sizes, drug loading, and surface charges, which are critical for treatment performance. Immunoreactivity and toxicity are other significant obstacles in neurotherapeutics (Table 1) [101]. Positively charged NPs improve cell absorption, and they can cause hemolysis and trigger an inflammatory response. In addition, MNPs such as AgNPs and AuNPs exhibit dose-dependent toxicity, and at high concentrations, there is a risk of oxidative stress and neural tissue damage [49,50,85]. However, the toxicity of MNPs can be minimized through a combination of strategies such as functionalization with antioxidant/biocompatible agents, surface charge modulation, size control, selective targeting mechanisms, and controlled release systems, which can ensure the safe use of MNPs in neurotherapeutic applications. The formation of the protein corona, a biomolecule layer adsorbed on the NP’s surface, can alter the distribution and reduce efficacy [103,104]. Regulatory and ethical issues also complicate the clinical translation of nanotherapy. Regulatory approval is delayed due to the lack of standardized guidelines for evaluating the pharmacokinetics, biological distribution, and long-term toxicity of NPs [105]. Ethical issues, especially the environmental impact and potential bioaccumulation of NPs, further highlight the need for thorough risk assessment [105,106]. Solving these limitations requires the development of more robust NP designs with improved stability, scalability, and safety profiles. Strategies such as surface functionalization, the precise control of NPs, and the use of biocompatible coatings are being explored to overcome these challenges. However, substantial research efforts and the development of standardized regulations ensure the successful transition of these technologies to clinical trials.

## 11. Application and Translational Aspects

NP-based therapies are progressing through various stages of development, with some technologies already advancing to clinical trials [107,108]. For example, liposomal formulations such as Doxil (liposomal doxorubicin) and Nanotherm are FDA-approved for cancer therapy, highlighting the feasibility of nanotechnologies for clinical applications. In the context of neurological disorders, liposomal nanocarriers functionalized with BBB-targeting ligands, such as transferrin and lactoferrin, are among the most advanced candidates, showing promise in preclinical studies for efficient drug delivery to the CNS. SLNs and PBNPs, including PLGA (poly-lactic-co-glycolic acid) systems, are also being evaluated in translational settings, with some reaching early-phase clinical trials for drug delivery in neurological diseases and brain tumors [109,110]. Despite these advances, several challenges persist. The regulatory framework for nanomedicine is still evolving, with a lack of harmonized guidelines for characterizing nanoparticle safety, efficacy, and pharmacokinetics. Issues such as batch-to-batch variability, scale-up production, and long-term biocompatibility require stringent evaluation. Additionally, the translation of nanotherapeutics involves overcoming practical challenges, including the high cost of manufacturing, the stability of nanoparticle formulations during storage, and ensuring patient accessibility. We have highlighted these barriers and emphasized strategies to address them, such as the development of standardized protocols for NP characterization and advancements in scalable cost-effective manufacturing techniques. Furthermore, interdisciplinary collaborations between researchers, clinicians, and regulatory agencies are essential to streamline the translation of nanotherapies from bench to bedside [107,108,111].

## 12. Conclusions and Future Perspectives

Progress in the treatment of neurological disorders has been substantial due to the advances in nanotherapeutics [112,113]. Nanotherapeutics is an advanced and expanding approach for treating neurological disorders with promising future prospects. In many cases, phytomolecule-encapsulated NPs are potentially used in the treatment of neurological disorders [114]. Researchers are exploring different therapeutic approaches both traditional and modern to identify the most suitable treatment options for individual patients [115]. PBNPs such as PGLA-based NPs have shown all characteristics for neuroprotective drug delivery in neurological disorders. The surface encapsulation of NPs with therapeutic agents or the encapsulation of drugs at nanodimensional size has been found to be a successful technique for non-invasive intranasal drug delivery and for penetrating the BBB. These nanoparticles with diagnostic agents can be utilized as theranostics for both the early detection or diagnosis of diseases, as well as for targeted patient-specific therapy. Intranasal administration, being the most effective pathway to cross the BBB, opens opportunities to develop NP-associated vaccines for neurological diseases and other neuroinfectious diseases. In the future, LBNP systems can be engineered to achieve the precise targeting of diseased neural tissues. SLNs and other nanostructured lipid carriers are prominent types of nanocarriers identified for drug delivery in neurological diseases and neuroinfectious diseases like meningitis.

In summary, the future holds great potential for advancing nanotherapeutic treatments for neurological disorders and neuroinfectious diseases. As research progresses, these therapies are expected to become increasingly precise, resulting in more effective solutions for conditions such as meningitis. To further advance nanotherapeutics for neurological disorders, several areas need to be explored. One crucial focus is the development of more targeted carrier systems for specific diseases, such as engineered lipid-based NP systems. Additionally, designing delivery systems capable of effectively targeting specific regions of the brain or CNS could significantly improve treatment outcomes. Another potential area involves the development of novel nanocarriers with high biocompatibility and low toxicity, ensuring safety while minimizing adverse side effects. Additionally, integrating multiple therapeutic agents within a single nanocarrier offers the potential for synergistic effects, enhancing treatment outcomes in patients with neurological disorders. The application of nanoparticle-based systems for targeted drug delivery in meningitis represents a significant and promising therapeutic advancement to combat both rapidly progressing and long-term complications of meningitis and other neurological disorders. The potential for nanotherapeutic approaches to transform the treatment of neurological disorders and neuroinfectious diseases, especially meningitis, is substantial. With sustained research and development, these innovative nanotherapeutic approaches will undoubtedly lead to increasingly effective treatments for these debilitating conditions, ultimately improving the quality of life of countless patients affected by neurological disorders.

## Figures and Tables

**Table 1:** Classification of nanoparticles based on their size, toxicity, composition, and applications.

Nanoparticle	Size (nm)	Toxicity	Composition	Application
Liposomes	50–900	Potential moderate cytotoxicity	Phospholipids (hydrophilic and hydrophobic)	Anticancer, breast cancer drug delivery, anti-inflammatory capabilities
Solid NPs	100–500	Non-toxic (surface charge)	Solid lipid (hydrophobic)	Major depressive disorder, anticancer, dopamine expression
Polymer NPs	100–900	Non-toxic	Polymer crosslinking agent (hydrophilic)	Inner ear disease treatment, colon cancer, drug release and bioavailability, diabetes
Dendrimer	3–20	Moderate toxicity	Oligosaccharides (hydrophilic, hydrophobic)	Cancer therapy, drug delivery
